# 1,6-Bis(prop-2-yn-1-yl­oxy)naphthalene

**DOI:** 10.1107/S1600536811027310

**Published:** 2011-07-16

**Authors:** Qin-wan Yang, Jingu Shi, Tao Pang

**Affiliations:** a Key Laboratory of Pesticide and Chemical Biology of the Ministry of Education, College of Chemistry, Central China Normal University, Wuhan 430079, People’s Republic of China

## Abstract

The title compound, C_16_H_12_O_2_, contains two prop-2-yn-1-yl­oxy groups attached to a naphthalene ring system at the 1- and 6-positions. The crystal packing includes an inter­molecular C—H⋯π inter­action between a terminal ethynyl H atom and an ethynyl group on a glide-related mol­ecule and another inter­action between an O-atom-linked methyl­ene H and an ethynyl group of a different glide-related mol­ecule.

## Related literature

For the preparation of the title compound, see: Srinivasan *et al.* (2006 ▶). For biological and commercial applications of naphthalene derivatives, see Morikawa & Takahashi (2004 ▶). 
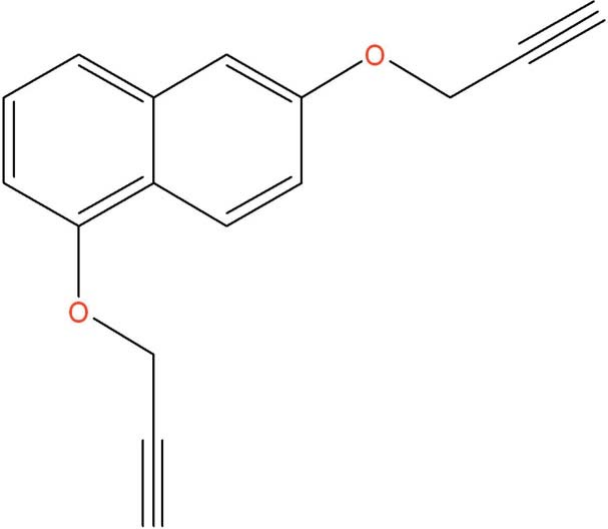


## Experimental

### 

#### Crystal data

C_16_H_12_O_2_
*M* *_r_* = 236.26
Monoclinic, 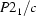

*a* = 5.1472 (9) Å
*b* = 10.3788 (19) Å
*c* = 23.409 (4) Å
β = 95.459 (3)°
*V* = 1244.9 (4) Å^3^
*Z* = 4
Mo *K*α radiation
μ = 0.08 mm^−1^
*T* = 298 K
0.16 × 0.12 × 0.10 mm

#### Data collection

Bruker SMART CCD area-detector diffractometer
7491 measured reflections
2306 independent reflections
1636 reflections with *I* > 2σ(*I*)
*R*_int_ = 0.118

#### Refinement

*R*[*F* ^2^ > 2σ(*F* ^2^)] = 0.051
*wR*(*F* ^2^) = 0.133
*S* = 1.02
2306 reflections
163 parameters
H-atom parameters constrained
Δρ_max_ = 0.15 e Å^−3^
Δρ_min_ = −0.15 e Å^−3^

### 

Data collection: *SMART* (Bruker, 1997 ▶); cell refinement: *SAINT* (Bruker, 1999 ▶); data reduction: *SAINT*; program(s) used to solve structure: *SHELXS97* (Sheldrick, 2008 ▶); program(s) used to refine structure: *SHELXL97* (Sheldrick, 2008 ▶); molecular graphics: *SHELXTL* (Sheldrick, 2008 ▶); software used to prepare material for publication: *SHELXTL*.

## Supplementary Material

Crystal structure: contains datablock(s) I, global. DOI: 10.1107/S1600536811027310/pk2330sup1.cif

Structure factors: contains datablock(s) I. DOI: 10.1107/S1600536811027310/pk2330Isup2.hkl

Supplementary material file. DOI: 10.1107/S1600536811027310/pk2330Isup3.cml

Additional supplementary materials:  crystallographic information; 3D view; checkCIF report

## Figures and Tables

**Table 1:** Hydrogen-bond geometry (Å, °) *Cg*1 and *Cg*2 are the centroids of the C1–C4/C9/C10 and C4–C9 rings, respectively.

*D*—H⋯*A*	*D*—H	H⋯*A*	*D*⋯*A*	*D*—H⋯*A*
C11—H11*B*⋯*Cg*1^i^	0.97	2.75	3.602 (2)	147
C14—H14*A*⋯*Cg*2^ii^	0.97	2.76	3.457 (2)	130

Symmetry codes: (i) 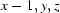; (ii) 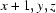.

**Fig. 1.**
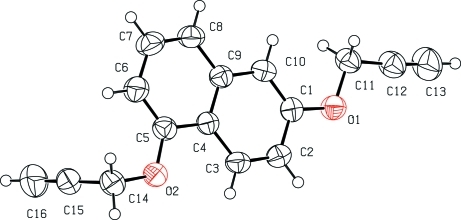
A view of the title compound with displacement ellipsoids drawn at the 50% probability level. H atoms are represented by spheres of arbitrary radius.

## supplementary crystallographic information

### Comment

Naphthalene derivatives have been extensively employed in many fields, and some possess important biological and commercial applications, including use as disinfectants, insecticides and auxin plant hormones, rooting agents and so on (Morikawa & Takahashi, 2004;).  The title compound was prepared by a rapid reaction between hydroxybenzene and prop-2-yn-1-yl-4-methylbenzenesulfonate with the introduction of sodium hydride (Srinivasan *et al.*, 2006).  Here we report the crystal structure of the title compound (Fig. 1).  X-ray analysis reveals that the crystal structure is stabilized by intermolecular non-classical C—H···π interactions.

### Experimental

The title compound was synthesized according to the literature procedure of Srinivasan *et al.* (2006).  Single crystals suitable for x-ray diffraction were prepared by slow evaporation of a solution of the title compound in petroleum ether: ethyl acetate (75: 1) at room temperature.

### Refinement

All H atoms were initially located in a difference map, but were constrained to idealized geometry.  Constrained bond lengths and isotropic displacement parameters: (C—H = 0.97 Å) and *U*_iso_(H) = 1.2 *U*_eq_(C) for methylene, and (C—H = 0.93 Å) and *U*_iso_(H) = 1.2*U*_eq_(C) for aromatic H atoms, and (C—H = 0.93 Å) and *U*_iso_(H) = 1.2*U*_eq_(C) for alkynyl H atoms

### Crystal data

**Table:**

C_16_H_12_O_2_	*F*(000) = 496
*M**_r_* = 236.26	*D*_x_ = 1.261 Mg m^−^^3^
Monoclinic, *P*2_1_/*c*	Mo *K*α radiation, λ = 0.71073 Å
Hall symbol: -P 2ybc	Cell parameters from 1759 reflections
*a* = 5.1472 (9) Å	θ = 2.6–24.9°
*b* = 10.3788 (19) Å	µ = 0.08 mm^−^^1^
*c* = 23.409 (4) Å	*T* = 298 K
β = 95.459 (3)°	Block, colorless
*V* = 1244.9 (4) Å^3^	0.16 × 0.12 × 0.10 mm
*Z* = 4	

### Data collection

**Table:**

Bruker SMART CCD area-detector diffractometer	1636 reflections with *I* > 2σ(*I*)
Radiation source: fine-focus sealed tube	*R*_int_ = 0.118
graphite	θ_max_ = 25.5°, θ_min_ = 2.2°
φ and ω scans	*h* = −6→6
7491 measured reflections	*k* = −12→12
2306 independent reflections	*l* = −27→28

### Refinement

**Table:**

Refinement on *F*^2^	Primary atom site location: structure-invariant direct methods
Least-squares matrix: full	Secondary atom site location: difference Fourier map
*R*[*F*^2^ > 2σ(*F*^2^)] = 0.051	Hydrogen site location: inferred from neighbouring sites
*wR*(*F*^2^) = 0.133	H-atom parameters constrained
*S* = 1.02	*w* = 1/[σ^2^(*F*_o_^2^) + (0.0446*P*)^2^] where *P* = (*F*_o_^2^ + 2*F*_c_^2^)/3
2306 reflections	(Δ/σ)_max_ = 0.001
163 parameters	Δρ_max_ = 0.15 e Å^−^^3^
0 restraints	Δρ_min_ = −0.15 e Å^−^^3^

### Special details

**Table:**

Geometry. All e.s.d.'s (except the e.s.d. in the dihedral angle between two l.s. planes) are estimated using the full covariance matrix. The cell e.s.d.'s are taken into account individually in the estimation of e.s.d.'s in distances, angles and torsion angles; correlations between e.s.d.'s in cell parameters are only used when they are defined by crystal symmetry. An approximate (isotropic) treatment of cell e.s.d.'s is used for estimating e.s.d.'s involving l.s. planes.
Refinement. Refinement of *F*^2^ against ALL reflections. The weighted *R*-factor *wR* and goodness of fit *S* are based on *F*^2^, conventional *R*-factors *R* are based on *F*, with *F* set to zero for negative *F*^2^. The threshold expression of *F*^2^ > σ(*F*^2^) is used only for calculating *R*-factors(gt) *etc*. and is not relevant to the choice of reflections for refinement. *R*-factors based on *F*^2^ are statistically about twice as large as those based on *F*, and *R*- factors based on ALL data will be even larger.

### Fractional atomic coordinates and isotropic or equivalent isotropic displacement parameters (Å^2^)

**Table:**

	*x*	*y*	*z*	*U*_iso_*/*U*_eq_	
C1	0.6519 (3)	0.40676 (17)	0.23456 (7)	0.0423 (4)	
C2	0.8336 (3)	0.30562 (16)	0.23889 (8)	0.0466 (5)	
H2	0.8254	0.2419	0.2108	0.056*	
C3	1.0218 (3)	0.29989 (16)	0.28383 (8)	0.0449 (5)	
H3	1.1412	0.2324	0.2859	0.054*	
C4	1.0385 (3)	0.39506 (15)	0.32738 (7)	0.0398 (4)	
C5	1.2313 (4)	0.39288 (17)	0.37514 (8)	0.0457 (5)	
C6	1.2356 (4)	0.48592 (18)	0.41649 (8)	0.0548 (5)	
H6	1.3595	0.4829	0.4481	0.066*	
C7	1.0524 (4)	0.58592 (19)	0.41099 (8)	0.0605 (6)	
H7	1.0583	0.6494	0.4391	0.073*	
C8	0.8668 (4)	0.59320 (18)	0.36603 (8)	0.0532 (5)	
H8	0.7474	0.6607	0.3635	0.064*	
C9	0.8560 (3)	0.49704 (16)	0.32270 (7)	0.0420 (5)	
C10	0.6626 (3)	0.50095 (16)	0.27556 (7)	0.0442 (5)	
H10	0.5421	0.5680	0.2724	0.053*	
C11	0.2933 (4)	0.50677 (18)	0.18126 (8)	0.0514 (5)	
H11A	0.3888	0.5864	0.1775	0.062*	
H11B	0.1976	0.5134	0.2149	0.062*	
C12	0.1124 (4)	0.48650 (19)	0.13036 (9)	0.0582 (6)	
C13	−0.0400 (5)	0.4787 (2)	0.09085 (10)	0.0813 (8)	
H13	−0.1625	0.4725	0.0591	0.098*	
C14	1.6025 (4)	0.2831 (2)	0.42217 (8)	0.0543 (5)	
H14A	1.6820	0.3672	0.4288	0.065*	
H14B	1.7370	0.2243	0.4119	0.065*	
C15	1.5032 (4)	0.23846 (19)	0.47507 (9)	0.0558 (5)	
C16	1.4286 (5)	0.2017 (2)	0.51684 (11)	0.0820 (8)	
H16	1.3683	0.1719	0.5506	0.098*	
O1	0.4707 (2)	0.40117 (12)	0.18760 (5)	0.0510 (4)	
O2	1.4033 (2)	0.29188 (12)	0.37524 (5)	0.0534 (4)	

### Atomic displacement parameters (Å^2^)

**Table:**

	*U*^11^	*U*^22^	*U*^33^	*U*^12^	*U*^13^	*U*^23^
C1	0.0397 (10)	0.0445 (10)	0.0427 (10)	−0.0044 (8)	0.0047 (8)	0.0026 (8)
C2	0.0490 (11)	0.0427 (11)	0.0486 (11)	−0.0011 (9)	0.0073 (9)	−0.0047 (8)
C3	0.0458 (11)	0.0389 (10)	0.0514 (11)	0.0049 (8)	0.0111 (9)	−0.0004 (8)
C4	0.0421 (10)	0.0377 (10)	0.0409 (10)	−0.0034 (8)	0.0102 (8)	0.0036 (8)
C5	0.0453 (10)	0.0441 (10)	0.0480 (11)	0.0010 (9)	0.0069 (9)	0.0027 (9)
C6	0.0553 (12)	0.0552 (12)	0.0517 (12)	0.0022 (10)	−0.0063 (9)	−0.0077 (9)
C7	0.0661 (13)	0.0500 (12)	0.0637 (13)	0.0027 (10)	−0.0027 (11)	−0.0177 (10)
C8	0.0582 (12)	0.0405 (11)	0.0601 (13)	0.0047 (9)	0.0025 (11)	−0.0049 (9)
C9	0.0440 (11)	0.0375 (10)	0.0452 (10)	−0.0030 (8)	0.0078 (8)	0.0014 (8)
C10	0.0438 (11)	0.0374 (10)	0.0519 (11)	0.0037 (8)	0.0070 (9)	0.0021 (8)
C11	0.0525 (12)	0.0435 (11)	0.0570 (12)	−0.0012 (9)	−0.0004 (10)	0.0032 (9)
C12	0.0550 (13)	0.0540 (13)	0.0639 (14)	−0.0013 (10)	−0.0029 (11)	0.0030 (10)
C13	0.0733 (16)	0.0845 (18)	0.0803 (17)	−0.0014 (13)	−0.0239 (14)	−0.0015 (13)
C14	0.0470 (11)	0.0582 (12)	0.0562 (12)	0.0077 (9)	−0.0033 (10)	0.0021 (9)
C15	0.0614 (13)	0.0526 (12)	0.0521 (13)	0.0025 (10)	−0.0008 (10)	0.0029 (10)
C16	0.0972 (19)	0.0841 (18)	0.0662 (16)	0.0053 (15)	0.0150 (14)	0.0144 (13)
O1	0.0486 (7)	0.0505 (8)	0.0524 (8)	0.0020 (6)	−0.0027 (6)	−0.0058 (6)
O2	0.0547 (8)	0.0556 (8)	0.0485 (8)	0.0138 (6)	−0.0022 (6)	−0.0024 (6)

### Geometric parameters (Å, °)

**Table:**

C1—C10	1.368 (2)	C8—H8	0.9300
C1—O1	1.3732 (19)	C9—C10	1.415 (2)
C1—C2	1.403 (2)	C10—H10	0.9300
C2—C3	1.362 (2)	C11—O1	1.425 (2)
C2—H2	0.9300	C11—C12	1.456 (3)
C3—C4	1.416 (2)	C11—H11A	0.9700
C3—H3	0.9300	C11—H11B	0.9700
C4—C9	1.413 (2)	C12—C13	1.157 (3)
C4—C5	1.423 (2)	C13—H13	0.9300
C5—C6	1.366 (2)	C14—O2	1.433 (2)
C5—O2	1.372 (2)	C14—C15	1.459 (3)
C6—C7	1.400 (3)	C14—H14A	0.9700
C6—H6	0.9300	C14—H14B	0.9700
C7—C8	1.354 (2)	C15—C16	1.149 (3)
C7—H7	0.9300	C16—H16	0.9300
C8—C9	1.420 (2)		
			
C10—C1—O1	124.83 (16)	C4—C9—C10	119.71 (15)
C10—C1—C2	120.12 (17)	C4—C9—C8	119.37 (17)
O1—C1—C2	115.05 (15)	C10—C9—C8	120.91 (16)
C3—C2—C1	120.58 (16)	C1—C10—C9	120.37 (16)
C3—C2—H2	119.7	C1—C10—H10	119.8
C1—C2—H2	119.7	C9—C10—H10	119.8
C2—C3—C4	121.04 (16)	O1—C11—C12	109.13 (15)
C2—C3—H3	119.5	O1—C11—H11A	109.9
C4—C3—H3	119.5	C12—C11—H11A	109.9
C9—C4—C3	118.18 (16)	O1—C11—H11B	109.9
C9—C4—C5	118.81 (16)	C12—C11—H11B	109.9
C3—C4—C5	123.01 (16)	H11A—C11—H11B	108.3
C6—C5—O2	124.88 (17)	C13—C12—C11	175.0 (2)
C6—C5—C4	120.59 (17)	C12—C13—H13	180.0
O2—C5—C4	114.53 (15)	O2—C14—C15	112.83 (15)
C5—C6—C7	119.56 (18)	O2—C14—H14A	109.0
C5—C6—H6	120.2	C15—C14—H14A	109.0
C7—C6—H6	120.2	O2—C14—H14B	109.0
C8—C7—C6	122.14 (18)	C15—C14—H14B	109.0
C8—C7—H7	118.9	H14A—C14—H14B	107.8
C6—C7—H7	118.9	C16—C15—C14	178.7 (2)
C7—C8—C9	119.51 (18)	C15—C16—H16	180.0
C7—C8—H8	120.2	C1—O1—C11	115.49 (13)
C9—C8—H8	120.2	C5—O2—C14	117.69 (14)
			
C10—C1—C2—C3	0.1 (3)	C3—C4—C9—C8	179.46 (16)
O1—C1—C2—C3	−179.48 (14)	C5—C4—C9—C8	−1.0 (2)
C1—C2—C3—C4	0.3 (3)	C7—C8—C9—C4	0.3 (3)
C2—C3—C4—C9	−0.6 (2)	C7—C8—C9—C10	179.20 (17)
C2—C3—C4—C5	179.87 (15)	O1—C1—C10—C9	179.34 (14)
C9—C4—C5—C6	1.7 (2)	C2—C1—C10—C9	−0.2 (3)
C3—C4—C5—C6	−178.81 (17)	C4—C9—C10—C1	−0.1 (2)
C9—C4—C5—O2	−178.55 (14)	C8—C9—C10—C1	−179.04 (17)
C3—C4—C5—O2	1.0 (2)	C10—C1—O1—C11	3.4 (2)
O2—C5—C6—C7	178.69 (17)	C2—C1—O1—C11	−177.02 (14)
C4—C5—C6—C7	−1.6 (3)	C12—C11—O1—C1	−179.26 (14)
C5—C6—C7—C8	0.8 (3)	C6—C5—O2—C14	0.0 (3)
C6—C7—C8—C9	−0.1 (3)	C4—C5—O2—C14	−179.77 (14)
C3—C4—C9—C10	0.5 (2)	C15—C14—O2—C5	74.8 (2)
C5—C4—C9—C10	−179.96 (15)		

### Hydrogen-bond geometry (Å, °)

**Table:**

Cg1 and Cg2 are the centroids of the C1–C4/C9/C10 and C4–C9 rings, respectively.

**Table:**

*D*—H···*A*	*D*—H	H···*A*	*D*···*A*	*D*—H···*A*
C11—H11B···Cg1^i^	0.97	2.75	3.602 (2)	147
C14—H14A···Cg2^ii^	0.97	2.76	3.457 (2)	130

Symmetry codes: (i) *x*−1, *y*, *z*; (ii) *x*+1, *y*, *z*.
